# Does anterior chamber-associated immune deviation (ACAID) play a role in posterior lamellar keratoplasty? Case report of a splenectomized patient

**DOI:** 10.1186/s12886-019-1114-1

**Published:** 2019-05-02

**Authors:** Deniz Hos, Friederike Schaub, Claus Cursiefen

**Affiliations:** 1 Department of Ophthalmology, University of Cologne, Faculty of Medicine and University Hospital Cologne, Cologne, Germany; 2 Center for Molecular Medicine Cologne (CMMK), University of Cologne, Cologne, Germany

**Keywords:** Corneal transplantation, Keratoplasty, Anterior chamber-associated immune deviation, ACAID, Descemet membrane endothelial keratoplasty, DMEK

## Abstract

**Background:**

It has been shown experimentally in rodents that removal of the spleen leads to increased rejection of corneal allografts after corneal transplantation (keratoplasty).

**Case presentation:**

Here, we report a unique case of a splenectomized patient with corneal endothelial dystrophy who underwent posterior lamellar keratoplasty. During follow-up of 4 years, we did not detect any signs of corneal allograft rejection.

**Conclusions:**

Our report indicates that an intact spleen is not necessary for allograft acceptance after posterior lamellar keratoplasty. To the best of our knowledge, this is the first report of a splenectomized patient receiving a (lamellar) corneal transplant.

## Background

Corneal transplantation (keratoplasty) is the most common and most successful form of transplantation worldwide. Graft survival rates are usually excellent with no need of HLA-matching, as the cornea belongs to the immune-privileged tissues of the body [1]. One condition that essentially contributes to this immune privilege is the so-called “anterior chamber-associated immune deviation” (ACAID), which leads to a systemic and antigen-specific suppression of immune responses when alloantigens are introduced into the anterior chamber [1]. Importantly, the induction of ACAID requires the participation of the spleen, and it has been shown experimentally in rodents that removal of the spleen prevents the development of ACAID and leads to increased rejection of corneal allografts after keratoplasty [2].

Until recently, corneal transplantation was usually performed as full-thickness penetrating keratoplasty (PK), where all corneal layers were replaced by a donor cornea. Recently, novel lamellar grafting techniques have been developed and the most commonly used technique for posterior lamellar keratoplasty is Descemet membrane endothelial keratoplasty (DMEK), where only the diseased Descemet membrane and endothelium are replaced. DMEK has become the standard procedure for the treatment of endothelial disorders such as Fuchs corneal endothelial dystrophy (FCED) and shows superior visual rehabilitation and significantly lower risk of allograft rejection when compared to PK [3]. However, allograft rejection does occasionally occur after DMEK, especially during the first two postoperative years [4]. Whether the mechanisms of ACAID are also responsible for the low rates of graft rejection in DMEK, is currently unknown. Furthermore, it is unknown whether splenectomy prevents the potential development of ACAID and leads to allograft rejection after DMEK.

## Case presentation

Here, we report a unique case of a 76-year old female patient with FCED who underwent DMEK surgery. Several years before, the patient’s spleen had to be removed because of an iatrogenic splenic injury during laparoscopy. The patient was generally immunocompetent and not unusually susceptible to infections. DMEK surgery without HLA-matching was performed without complications. Postoperatively, the patient received topical corticosteroids (prednisolone acetate 1% 5 times daily), which was slowly tapered and stopped after 2 years, antibiotics (Ofloxacin 3 times daily for 2 weeks) and lubricants. The patient was followed regularly by slit-lamp biomicroscopy and visual acuity and endothelial cell densities were determined. During follow-up, we did not detect any signs of allograft rejection. Visual acuity was 20/25 at 3 months and 20/20 at 1, 3, and 4 years postoperatively. Donor endothelial cell density was 2553 cells/mm^2^ preoperatively, 1779 cells/mm^2^ at 3 months, 1609 cells/mm^2^ at 1 year, 1377 cells/mm^2^ at 3 years, and 1274 cells/mm^2^ at 4 years postoperatively. Thus, endothelial cell loss rates were comparable to rates reported for eyes without rejection [5].

## Conclusions

Experimentally, an intact spleen is a prerequisite for ACAID, and removal of the spleen in rodents leads to 90–100% corneal allograft rejection after PK [1, 2]. Whether this also holds true in humans and in lamellar keratoplasty, has not been shown so far. To our knowledge, this is the first report of a splenectomized patient receiving a (lamellar) corneal transplant. The fact that we did not observe any signs of allograft rejection indicates that an intact spleen is not necessary for allograft acceptance, at least after DMEK.

Interestingly, work from Hori and Streilein in mice have shown that graft rejection is significantly reduced when chimeric grafts (consisting of syngeneic epithelium and allogeneic stroma-endothelium) are used [6]. Such chimeric grafts might be protected from graft rejection not by an active suppression of immune responses but rather by immunological ignorance [7]. The situation of our patient who received a posterior lamellar allograft without transplantation of the anterior cornea has similarities with such a chimeric graft, although in DMEK only the Descemet membrane and not the additional stroma is of allogenic origin. Thus, it might be possible that the absence of graft rejection in our patient might be attributed not to ACAID but to immunological ignorance. In this context, it might be very interesting to evaluate whether chimeric grafts in splenectomized mice would result in graft rejection or not.

As mentioned, our patient received topical corticosteroids for the first 2 years after keratoplasty, which is known to significantly reduce the risk of allograft rejection after keratoplasty [8, 9]. After termination of topical corticosteroids, the risk allograft rejection might be increased [9], although we did not observe any signs of allograft rejection in our patient so far. Nevertheless, additional follow-up will have to confirm that allograft rejection is not occurring in our patient, also considering that the risk of allograft rejection after DMEK is generally lower when compared to PK [3, 4].

## Abbreviations

ACAID: anterior chamber-associated immune deviation
DMEK: Descemet membrane endothelial keratoplasty;
FCED: Fuchs corneal endothelial dystrophy
HLA: human leucocyte antigen
PK: penetrating keratoplasty
